# Differential scanning fluorimetry followed by microscale thermophoresis and/or isothermal titration calorimetry as an efficient tool for ligand screening

**DOI:** 10.1007/s12551-025-01280-3

**Published:** 2025-02-13

**Authors:** Maria Winiewska-Szajewska, Jarosław Poznański

**Affiliations:** Institute of Biochemistry and Biophysics PAS, Pawinskiego 5a, 02-106 Warsaw, Poland

**Keywords:** Differential scanning fluorimetry (DSF), Microscale thermophoresis (MST), Isothermal titration calorimetry (ITC), Binding affinity, Ligand screening, Protein–ligand interaction

## Abstract

Various biophysical and biochemical techniques have been developed to measure the affinity of interacting molecules. This review analyzes the combination of three methods: differential scanning fluorimetry as the initial high-throughput screening technique and microscale thermophoresis and isothermal titration calorimetry as complementary methods to quantify binding affinity. The presented work is the first to detailed compare the strengths and flaws of these three specific methods, as well as their application possibilities and complementarity. The fundamentals of these methods will be covered, including the most often-used models for characterizing observable phenomena and an emphasis on methods for analyzing data. A comprehensive review of numerous approaches to data analysis found in the literature is additionally provided, with the benefits and drawbacks of each, as well as the pitfalls and related concerns. Finally, examples of different systems will be presented, and methods used and some discrepancies in results will be described and discussed.

## Introduction

Numerous biophysical and biochemical techniques have been developed to measure the affinity of interacting molecules and provide information on the binding mechanism and kinetics. All of these methods are based on specific approaches, ranging from semi-quantitative biochemical assays to procedures that assess binding kinetics (e.g., Surface Plasmon Resonance—SPR) or the enthalpy of interaction (e.g., Isothermal Titration Calorimetry—ITC). Each method encloses advantages and disadvantages regarding sample and time consumption, complexity, and reliability, so choosing one universal technique optimal for any system remains impossible. However, using the combinations of different biophysical techniques allows us to correctly and efficiently estimate binding affinity.

Many high-throughput affinity screening techniques for testing a large number of compounds in a short time burden and finding the most promising ones are routinely used. Starting with ELISA-based methods (Ozgul et al. 2019) or other assays when optimized for specific targets also can be high-throughput (e.g. FRET or FliK assays (Getlik et al. 2012; Liao et al. 2015)). However, also affinity-selection methods—usually coupled with mass spectrometry (AS-MS) (Prudent et al. 2021), DEL: DNA-encoded libraries (Yuen and Franzini 2017; Gironda-MartíNez et al. 2021), and also described in detail in this review Differential Scanning Fluorimetry (DSF) or Thermal Shift Assays (TSA) and this method in a cellular format called the cellular thermal shift assay (CETSA) (Jafari et al. 2014).

Unfortunately, high-throughput methods usually have limitations in terms of sensitivity and scope of the information collected, but there is also a specific risk of false positive or negative hits. So, all identified interactions should always be independently confirmed, preferably by quantitative determination of the binding affinity. Many biophysical methods can currently be employed to measure binding affinities directly. Surface Plasmon Resonance (SPR) has been used for the last three decades to analyze the kinetics of biomolecular interactions (Patching 2014; Nguyen et al. 2015). Other surface-based, label-free technologies are Biolayer Interferometry (BLI) (Perillat et al. 2009) and Grating-coupled interferometry (GCI) (Kozma et al. 2009). A wide range of NMR methods are also dedicated to determining binding affinities of ligands toward proteins (Fielding 2003). Among an increasing number of Mass spectrometry applications, affinity measurements are one of them (Erba and Zenobi, 2011; Schulte et al., 2023). Another group of methods to estimate the dissociation constants of protein–ligand complexes is fluorescence-based approaches (Brown and Royer 1997), like Fluorescence anisotropy (FA) and polarization (FP) (Owicki 2000) or fluorescence correlation spectroscopy (FCS) (Thompson et al. 2002). However, changes in fluorescence intensity (intrinsic or that of attached fluorophore) upon binding are also used to quantify binding (Brown and Royer 1997; Mocz and Ross 2013). Any other spectroscopic methods, such as Circular dichroism (Rodger et al., 2005), may be applied whenever ligand binding is reflected in a measurable change in signal. The radioisotopic method is another technique that has been used for over 40 years (Hart and Greenwald, 1979; Bosworth and Towers 1989; Mccrea and Herzog 2000). Also, all separative methods like chromatographic techniques (Sebille et al. 1990), equilibrium dialysis (ED) (Kariv et al. 2001), Capillary electrophoretic techniques (Tanaka and Terabe 2002), and others (Vuignier et al. 2010) should also be mentioned. In this review, we focus on two methods, the first known as the gold-standard technique for studying binding processes—isothermal titration calorimetry (ITC), and the second relatively new but gaining popularity very fast—microscale thermophoresis (MST). At this point, however, it is worth noting that there is no strict division into screening methods and those that allow direct determination of binding constants. Thus, some of the methods presented in the second part (such as NMR, SPR, and various fluorescence-based methods) with certain modifications are also used as screening methods (Moore 1999; Owicki 2000; Navratilova and Hopkins 2010; Dalvit and Vulpetti 2019).

**Fig. 1:**
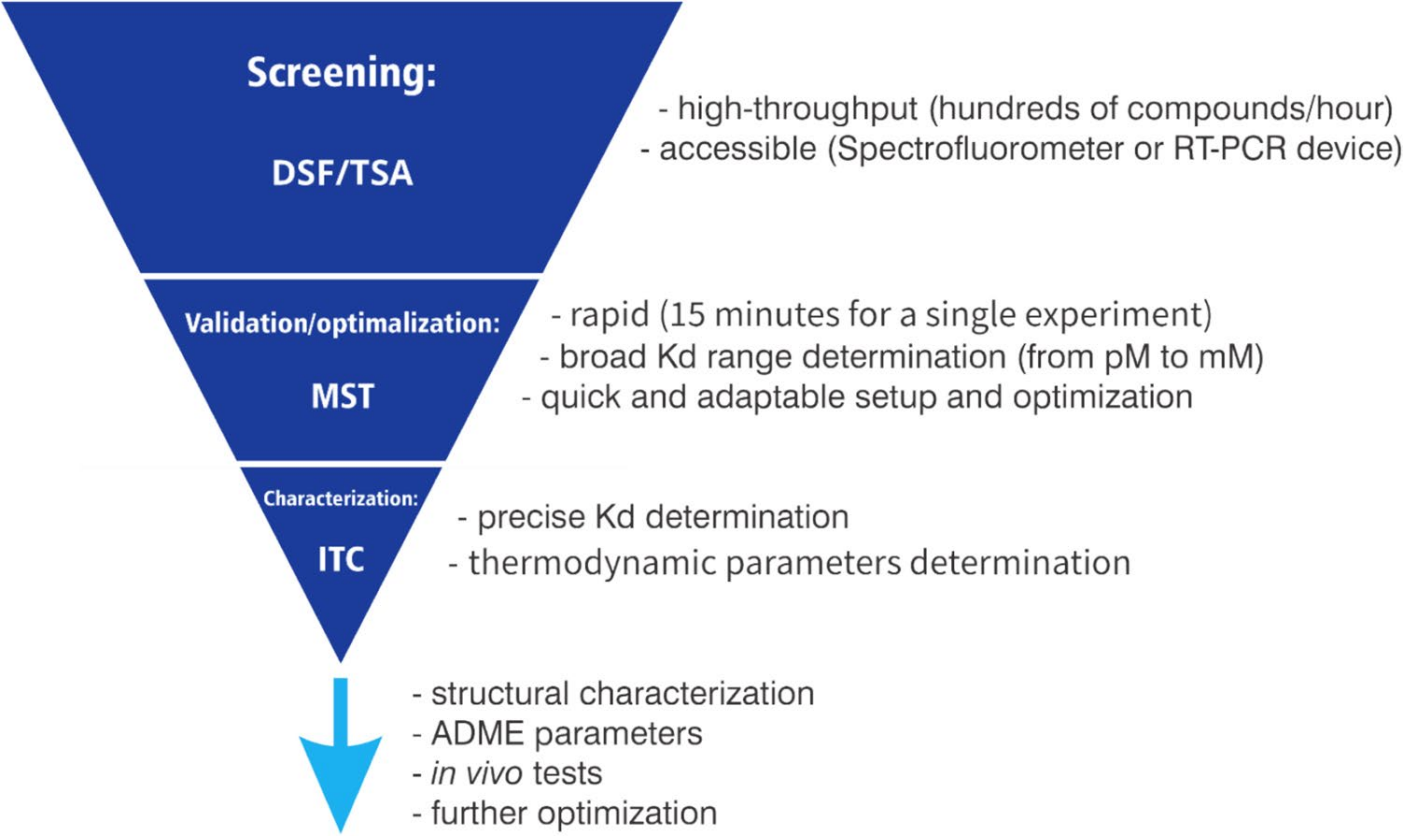
Proposed hit screening procedure using DSF followed by ITC/MST methods with main advantages of these approaches and marked further steps for drug design

Most of these methods require dedicated hardware and some competencies in data analysis. It may also be problematic to optimize experimental conditions without knowledge about the studied interaction. However, one of the most effective approaches to identifying promising ligands remains to combine basic screening methods with quantitative ones. We found that the already proposed procedure with preliminary screening, validation and characterization stages (Mashalidis et al. 2013) is very effective, regardless of the methods proposed at each stage, but the combination proposed in this review additionally facilitates and makes the procedure more accessible (Fig. 1).

This review focuses on the efficient combinations of three biophysical techniques for detecting and quantifying protein–ligand interactions, DSF followed by MST and ITC. While the rest of the paper will focus just on these three strategies, Table 1 shows the most commonly used biophysical techniques with their advantages and limitations.

**Table 1:** Comparison of most common biophysical method for quantifying ligand binding

Method	Approach	Information	Advantages	Limitations	Ref
Thermal shift assay(TSA) or Differential scanning fluorimetry (DSF)	Label-free	Screening; Binding- yes/no information	High throughput, widely available equipment, easy to use Low sample consumption	False positives and negatives	(Gao et al. 2020)
	With fluorescent dye			Requires a dye, many false positives and negatives	(Bai et al. 2019; Gao et al. 2020)
Other thermal shift methods	Differential scanning calorimetry (DSC)	Binding- yes/no information	Highly sensitive method. Label-free	Low throughput High sample consumption	(Gill et al. 2010; Llowarch et al. 2023)
	Monitored with the aid of CD,UV–VIS, NMR CD		Label-free. Low sample consumption	Limited throughput	(Greenfield 2006)
Surface Plasmon Resonance (SPR)		Binding affinity, kinetic data	Label-free.Low sample consumption, sensitive method, can be high throughput	Protein needs to be immobilized, requires complex system optimization. High cost	(Navratilova and Hopkins 2010; Patching 2014; Nguyen et al. 2015)
Other surface-based methods	Biolayer Interferometry (BLI)		Label-free. Low sample consumption, sensitive method, more high throughput than SPR	Protein needs to be immobilized. Limited application for tight binders and some molecules. High cost	(Perillat et al. 2009; Jug et al. 2024)
	Grating-coupled Interferometry (GCI)			Protein needs to be immobilized. High cost	(Kozma et al. 2009)
Nuclear magnetic resonance (NMR)-based method	Ligand-observed NMR	Binding affinity, Screening—identification of the specific components binding the target	High throughput. All molecules can be tested simultaneously. No isotopically labeled protein needed	High sample consumption. Limited to fragments with fast exchange with the target. High cost, requires expertise	(Craik and Wilce 1997; Moore 1999; Carlomagno 2005)
	Protein-observed	Binding affinity, binding site information	All molecules can be tested simultaneously	High sample consumption. Limited throughput, Isotopically labelled protein needed. Complex NMR assignment and structure determination. High cost, requires expertise	(Craik and Wilce 1997; Moore 1999; Fielding 2003; Dalvit and Vulpetti 2019)
Mass spectrometry (MS)-based methods	Native MS	Binding affinity, screening	Label-free. High sensitivity, selectivity, rapid and simultaneous measurement of multiple ligands	Limited applicability to biological systems. High cost, requires expertise	(Hofstadler and Sannes-Lowery 2006; Vivat Hannah et al. 2010)
	Ligand-observed MS (including AS-MS)	Binding affinity, screening	Label-free. High sensitivity, selectivity. High throughput. All molecules can be tested simultaneously	At least two steps required. High cost, requires expertise	(Chen et al. 2015b; Prudent et al. 2021; Simon et al. 2021)
	Hydrogen- deuterium Mass Spectrometry (HDX-MS)	Binding yes/no and binding site information	Label free, sensitive	High sample consumption, easy to misinterpreted. High cost, requires expertise. Low throughput	(Chalmers et al. 2011)
Isothermal titration calorimetry (ITC)		Binding affinity, Thermodynamic parameters	Label-free. Low cost	High sample consumption. Limited throughput	(Freyer and Lewis 2008; Bastos et al. 2023)
Microscale thermophoresis (MST)		Binding affinity	Low sample consumption, Fast, Easy to use and optimize	Average cost. Usually requires a dye	(Jerabek-Willemsen et al. 2014)
Fluorescence-based approaches	Fluorescence anisotropy (FA) and polarization (FP)	Binding affinity, screening, kinetic data	Low sample consumption. High throughput, easy to use	Average cost. Requires a dye	(Owicki 2000; Holdgate and Hemsley 2021)
	Fluorescence correlation spectroscopy (FCS)	Binding affinity, kinetic data	Low sample consumption. High sensitivity	Requires a dye and/or optimization for system. Average cost, requires some expertise	(Thompson et al. 2002; Yu et al. 2021)
	Förster resonance energy transfer (FRET)	Binding affinity, screening, kinetic data	Low sample consumption. High sensitivity. High throughput	High cost. Requires dye and optimization for the system	(Okamoto and Sako 2017; Deleeuw et al. 2021; Liao 2023)
	Fluorescence intensity (FI)	Binding affinity, kinetic data	Low sample consumption	May require a dye, false positives and negatives. Limited applicability	(Hovius et al. 2000)
Radioisotopic methods		Binding affinity, screening	Fast, easy to use	High cost. Requires radioisotope labelling	(Wu and Liu 2005)
Separative methods		Binding affinity	The most direct methods for binding affinity measurement	Type of detection is limiting factor Low throughput, prone to errors	(Sebille et al. 1990)
Crystallography		Binding- yes/no information, detailed binding site information	Provide a detailed view of the interaction	High sample consumption. High cost, requires expertise	(Mcnae et al. 2005)

## Differential scanning fluorimetry

Differential scanning fluorimetry (DSF) or thermal shift assays (TSA), a high-throughput method for screening compounds, was initially proposed over 20 years ago (Pantoliano et al. 2001; Lo et al. 2004). However, the fluorescence-monitored thermal denaturation of proteins has been commonly used since at least the 1960s (Steiner and Edelhoch 1962). The great advantage of this method is the accessibility of the required equipment in most laboratories; the minimal hardware setup requires the fluorimeter with a temperature-controlled cuvette holder. The experiments are easy to set up. Moreover, this method may sample an extensive affinity range, correctly identifying high-affinity ligands. The binding affinities of tested compounds are estimated based on the idea that the ligand binding will stabilize the protein, therefore making the protein unfold at a higher temperature. The increase in the melting temperature (T_m_) of the protein–ligand complex relative to a protein in the absence of a ligand is called thermal shift (ΔT_m_). For closely related rigid ligands that similarly bind to the target protein, for which the enthalpic contribution to the binding predominates the entropic and heat capacity effects, the thermal shift can be used to rank ligands according to their affinities. However, no general correlation exists between the ligand-induced increase of the target thermal stability (ΔT_m_) and the ligand binding affinity. The thermal shift effect represents the ligand binding affinity at the melting temperature, which may substantially differ from the affinity at the reference temperature (usually T_ref_ = 25 °C). The temperature-dependence of entropy-enthalpy balance (both for the protein unfolding and ligand binding), additionally affected by the heat capacity effect, could, in extreme cases, completely alter the screening results. Thus, ligands with close affinity to the same target at T_ref_ may cause different thermal stabilization, ΔT_m_. Consequently, ligands that display the same ΔT_m_ can differ in their binding affinity at T_ref_. Using the DSF method, one must also accept the occurrence of false negatives for enthalpy-driven binding with a large unfavourable entropic or heat capacity contribution: ΔG_bind_(T_ref_) < 0 and ∂ΔG_bind_/∂T > 0 or false positives for entropy-driven binding almost balanced at T_ref_ by unfavourable binding enthalpy: ΔG_bind_(T_ref_) ≈ 0, and ∂ΔG_bind_/∂T < 0). So, the application of DSF should be restricted to screenings focused on identifying strong binders when no ligand affinity ranking is required. It is worth adding, however, that despite the qualitative nature of the DSF experiment, some thermodynamic parameters describing ligand binding (i.e., could be roughly estimated from the dependence of T_m_ on the ligand concentration (Cimmperman et al., 2008).

The thermal shift can also be measured using alternative techniques like Differential Scanning Calorimetry (DSC), Nuclear magnetic resonance spectroscopy (NMR), Circular Dichroism (CD), or other spectrometric methods (e.g., UV–Vis), all of which have been successfully used for decades (Pace and Mcgrath 1980; Brandts and Lin 1990; Straume and Freire 1992; Bouvier and Wiley 1994; Morton et al. 1995; Cahen et al. 2000). However, DSF stood out from these other techniques by employing high-throughput screening as an alternative to testing one condition at a time.

Real-time polymerase chain reaction (RT-PCR) equipment is commonly accessible in standard molecular biology labs. Such a device enables measuring DSF using a hydrophobic fluorescent dye that binds preferably to an unfolded protein, which may be called extrinsic fluorescence. Any dye whose emission is compatible with the PCR-reader optical system can be used as the reporter—up to now, this is the most frequently used DSF approach (Niesen et al. 2007). However, over time, spectrofluorometers monitoring intrinsic fluorescence were modified to measure simultaneously more samples in a single run, and even dedicated systems have been built (Strutz 2016).

### Extrinsic fluorescence

The fluorescence of dyes that can be used for DSF is highly sensitive to the environment, being quenched in aqueous solution when the protein target is folded, compared to a high level when the dye is protected from the solvent due to its binding to solvent-exposed hydrophobic regions of an unfolded protein (Pantoliano et al. 2001). The most common dye used in DSF is SYPRO Orange; however, many other commercial dyes are available in the market (Hawe et al. 2008). Such an approach is valid only under the assumption that the folded state of the tested protein is moderately hydrophobic. Hence, the fluorescent dye binds preferably to an unfolded form, in which hydrophobic residues constituting the hydrophobic core become solvent-exposed. Thus, a larger hydrophobic surface is accessible to the dye. However, a reverse situation may also occasionally occur when dye binds stronger to the native protein, as it was reported for ANS binding by Human Serum Albumin (Celej et al. 2005). Moreover, neither the protein nor the fluorescent dye should react with other components or cofactors. Some virtually uncontrolled temperature-dependent effects, including oligomerization, aggregation, partial unfolding (e.g., a stepwise unfolding of multidomain proteins), or structural rearrangements, may significantly hinder binding effects or even make data analysis impossible.

### Intrinsic fluorescence

The DSF experiment can use the fluorescence of the natural fluorophores of protein, which are aromatic sidechains of phenylalanine (Phe), tryptophan (Trp), and tyrosine (Tyr). The fluorescence of tyrosine and tryptophan residues is preferably used due to the high quantum efficiency. With excitation at 280 nm, one obtains the emission spectrum for both Trp and Tyr. The fluorescence yield of particular residues varies depending on their microenvironment, which usually differs for the native and unfolded state of a protein (Weber 1960; Konev 1967; Demchenko 2013). The thermal denaturation is usually monitored by the fluorescence intensity of tyrosine (~ 330 nm) (Van Mierlo and Steensma 2000; Winiewska et al. 2015), the whole emission spectrum (ZoldáK et al. 2004), or the 350 nm/330 nm intensity ratio (Boland et al. 2018; Real-Hohn et al. 2020; Le et al. 2022). However, alternative approaches (e.g., λ_max_ shift (Suh and Savizky 2012)) can also be used. While such methods allow the study of protein in its natural untagged form, the other approach, tagging with a fluorescent protein (GFP), does not require an external fluorescent reporter, which is also referred to as intrinsic fluorescence (Moreau et al. 2010). However, the size of the GFP, possible effects on target protein stability, and putative interactions with the ligands make such a DSF variant less universal.

### Analyzing thermal denaturation data—T_m_ determination

**Fig. 2:**
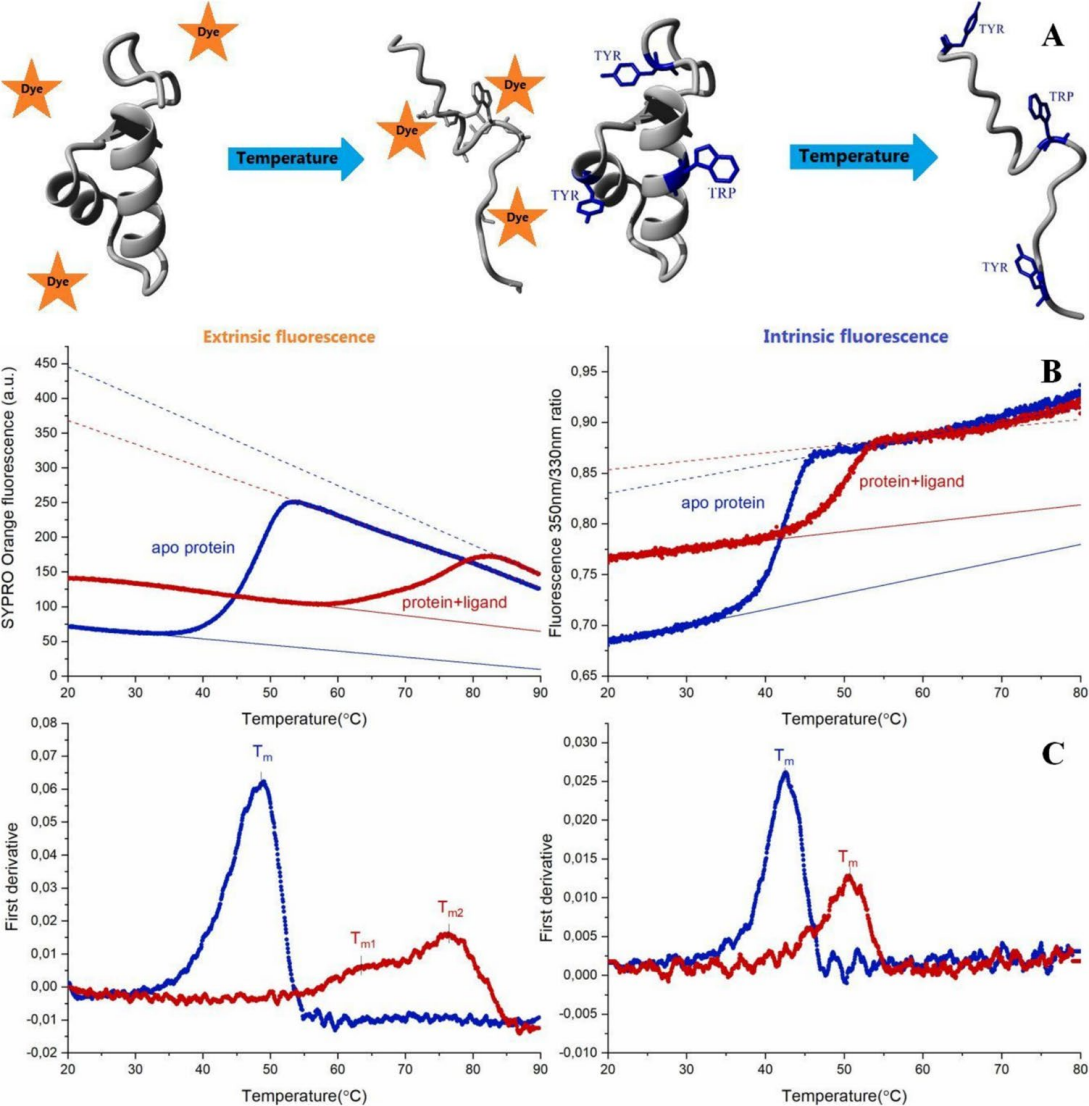
Differential scanning fluorimetry. **A** Cartoon representing the native and unfolded states of protein for a method using extrinsic (left panel) and intrinsic (right panel) fluorescence, **B** examples of raw data obtained for extrinsic (data of Fe^2+^ binding to FTO partly adapted from (Marcinkowski et al. 2021)) and intrinsic experiments (data of 4,5,6-tribromo-1H-benzotriazole binding to hCK2α adapted from (Czapinska et al. 2021)), and **C** their first derivatives

**Fig. 3:**
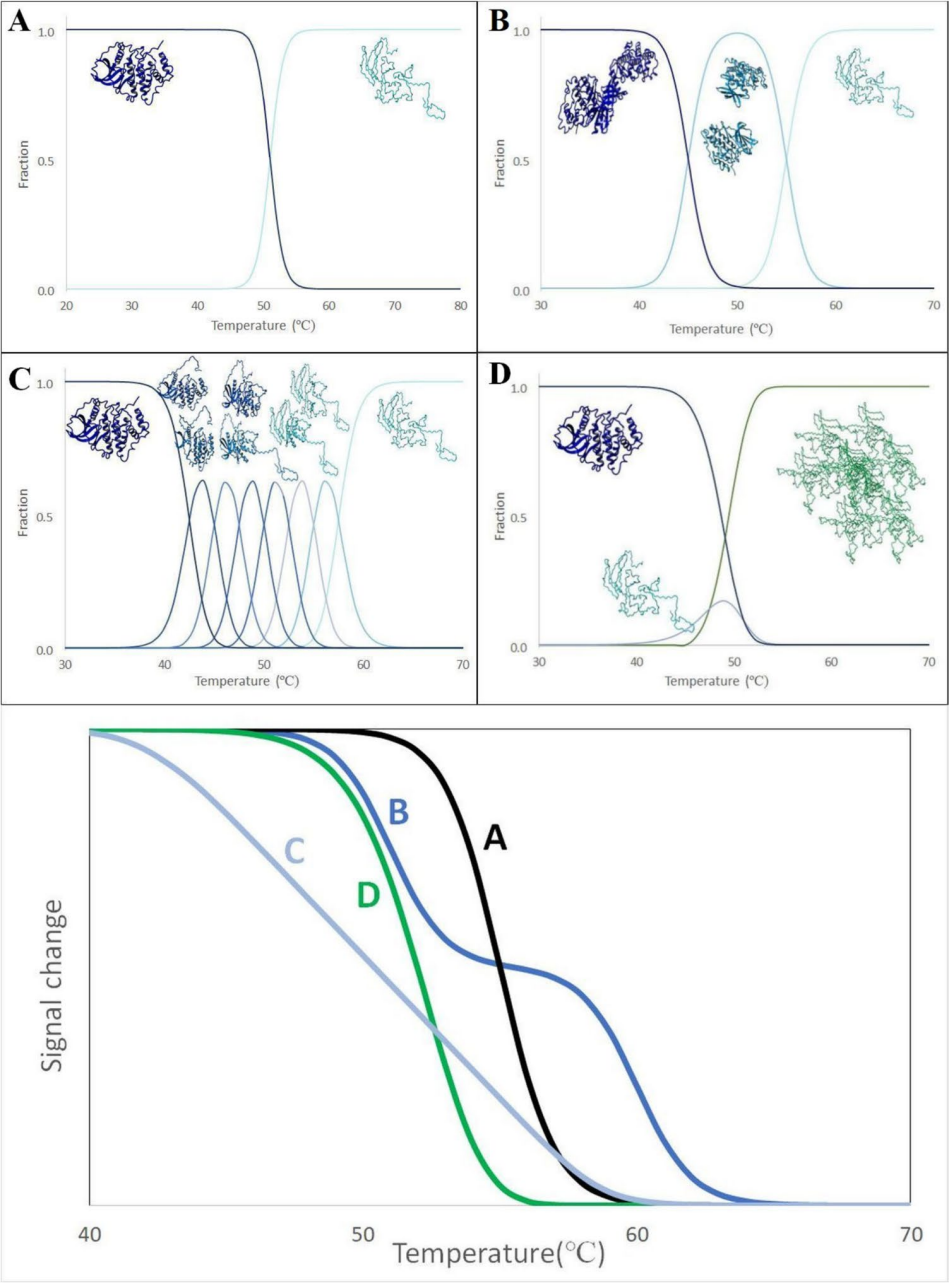
Simplified models of protein unfolding. Cartoon representing different models of protein denaturation and changes in the fraction of particular states with temperature. A) Two-state transition. B) Three-state transition. C) Sequential unfolding. D) Two-state unfolding followed by aggregation. In the bottom, the theoretical signal change with the unfolding (simplified with the assumption that signal change with fraction evenly) A-for simple two-state transition with Tm = 55℃; B-for three-state transition with Tm_1_ = 51℃ and Tm_2_ = 60℃; C- for multi-states sequential transition with melting temperatures ranging between 42.5℃ and 57.5℃ and D- for two-state transition with Tm = 55℃ followed by aggregation

Several alternative methods are used to analyze the thermal denaturation data obtained from DSF. The most straightforward and commonly used approach that does not require any additional assumptions about the thermodynamic model of the target protein unfolding (i.e., model-free approach) is to calculate the apparent midpoint transition temperature (T_m_) as the temperature at which the extremum of the first derivative appears (Fig. 2C). This method is used in various software for DSF data analysis (Wang et al. 2012; Rosa et al. 2015; Sun et al. 2020). A similar method that can be applied in the case of extrinsic fluorescence, also unrelated to any particular thermodynamic model, is determining T_m_ by calculating the temperature at which the fluorescence reaches 50% of the highest intensity (Sun et al. 2020). A typical temperature-dependent fluorescence follows a sigmoidal two-state transition (Fig. 3A or in the bottom-line A), so the alternative method is to apply a non-linear least squares algorithm to fit either a Boltzmann distribution or, preferably, the appropriate thermodynamic two-state model (i.e., folded–unfolded) to the experimental data. Such an analysis can be done with commercial (Schulz et al. 2013; Rosa et al. 2015; Lee et al. 2019) or hand-made (Eftink 1994; Pantoliano et al. 2001; Ericsson et al. 2006; Winiewska et al. 2015; Marzec et al. 2020) software.

Generally, the apparent T_m_ value determined with a model-free approach should be close to that estimated with the formal analysis based on the two-state model. However, in some cases, these two values may differ substantially. The two-state model provides more reliable estimates of the T_m_ whenever no multiple transitions are expected. Any discrepancy between these two approaches indicates unfolding processes associated with low enthalpy and/or large heat capacity change for a single transition process. An additional benefit of the formal analysis is that the two-state model also estimates the unfolding enthalpy and accompanied heat capacity.

However, many proteins do not undergo straightforward reversible two-state unfolding. In general, protein unfolding should always be regarded as a multistep process, even if intermediate states are only barely populated (Freire and Murphy 1991). Recently, Zimm–Bragg theory (Zimm and Bragg 1959) was proposed as a more realistic model for a protein unfolding process (Seelig and SchöNfeld 2016). While differences between various approaches proposed in the literature have little impact in the case of comparative binding studies, in some cases, the two-state model clearly cannot be employed. Some of the most common simplified unfolding patterns are presented in Fig. 3. Firstly, it is not difficult to imagine that larger, oligomeric proteins unfold through more stable intermediate steps (Fig. 3B); for example, dimer may initially dissociate into monomers. That may be reflected in two separated transitions (Fig. 3 in the bottom—line B), which are relatively easy to analyze using a formal three-state model of either independent or sequential unfolding (IrúN et al. 2001; Campos et al. 2004; Harder et al. 2004; Zheng and Yang 2010). However, when the melting profile of the intermediate overlaps with that of the native or the unfolded form, or the intermediate fraction is low, the two transitions may be unnoticeable. The number of intermediate stages can also be much larger, and cooperative interactions of protein domains will lead to an unfolding in a sequential manner (Fig. 3C). As a result, we will not observe the visible separation of each stage but rather the broadening of the transition curve (Fig. 3 Bottom—line C). In such cases, other approaches can be applied. There are sequential models (Seelig and SchöNfeld 2016) or, like in the DSC data analysis, a model described as non-two-state, in which a simple numeric approach is used to consider that data does not follow a two-state model of transition, in which an additional scaling parameter is added (DSC Data Analysis in Origin 1998). Another often observed effect is the aggregation of protein occurring in the unfolded state (Fig. 3D). The existence of irreversibility, by definition, is foreclosed using models based on equilibrium thermodynamics. Fortunately, a few models that deal with irreversible phenomena have already been proposed (Sanchez-Ruiz 1992; Kreimer et al. 1995; Gozdek et al. 2008). In most methods, intensive aggregation is usually reflected in signal output. The exception may be the 350 nm/330 nm fluorescence ratio, where intensities at both wavelengths may decline similarly, leading to the disregarded ratio change. However, new devices for label-free DSF can also monitor the aggregation phenomena by measuring back-reflection. When unfolding and aggregation occur together, the signal may transition at a lower temperature than when unfolding alone, as seen in Fig. 3E. That is another crucial reason for correctly recognizing such an event.

However, when the estimate of the melting temperature serves only for the screening procedure, for which the precise determination of the T_m_ is not critical, the application of a model-free approach seems sufficient. Otherwise, the T_m_ must be estimated using a formal model considering the contribution of all possible physical phenomena (e.g., enthalpy, heat capacity, aggregation rate, irreversibility).

### The thermodynamics of protein unfolding

There is still no consensus about the best model to describe thermal denaturation data. However, the thermodynamic basis of protein unfolding (focusing on the simplest two-state model) and ligand contribution must be addressed shortly.

Each microstate of the protein is characterized by the standard Gibbs free energy G^0^, and its occupancy (probability) under reversible equilibrium conditions is proportional to ${e}^{\frac{{-G}^{0}}{RT}}$. The simplest two-state model assumes a dynamic temperature-dependent equilibrium between two macro states (e.g., conformation, binding, ionic form), each possibly representing several indistinguishable microstates of a comparable G^0^. So, any reversible “steady-state” process, including protein folding/unfolding balance, can be described at a given temperature by the equilibrium constant, K, which, in turn, is related to the "standard" Gibbs free energy difference between these two states, ΔG^0^ (1).

1 $$K={e}^{\frac{{-\Delta G}^{0}}{RT}}; \quad \Delta {G}^{0}=\Delta {H}^{0}-T\Delta {S}^{0}$$

The “standard” parameters of Gibbs free energy (ΔG°), enthalpy (ΔH°), and entropy (ΔS°) refer to the hypothetical standard state in which all components in initial and final states are at 1 molar concentration (or activity). ΔH and ΔH° are practically identical under most conditions, but ΔS and ΔS° may significantly differ due to the extensive concentration-dependent entropy of mixing (Atkins and De Paula 2014).

Both changes in enthalpy and entropy are temperature-dependent according to the following equations:

2 $$\Delta H\left(T\right)=\Delta H\left({T}_{ref}\right)+\underset{{T}_{ref}}{\overset{T}{\int }}\Delta CpdT$$

3 $$\Delta S\left(T\right)=\Delta S\left({T}_{ref}\right)+\underset{{T}_{ref}}{\overset{T}{\int }}\frac{\Delta Cp}{T}dT$$

Assuming that over a limited temperature range, ΔCp does not significantly vary, the above equations can be formally integrated to give the temperature dependence of ΔH and ΔS relative to arbitrarily selected reference temperature (T_ref_):

4 $$\Delta H\left(T\right)\cong \Delta H\left({T}_{ref}\right)+\Delta {C}_{p}\cdot (T-{T}_{ref})$$

5 $$\Delta S\left(T\right)\cong \Delta S\left({T}_{ref}\right)+\Delta {C}_{p}\cdot \text{ln}(\frac{T}{{T}_{ref}})$$

In the simplest two-state unfolding model, the equilibrium between folded and unfolded states may be described as:

6 $${K}_{u}=\frac{[U]}{[F]}$$

where [U] and [F] indicate the populations of protein unfolded and folded state, respectively. Melting temperature or midpoint transition temperature (T_m_) is defined as the temperature at which both the folded and unfolded states are equally populated; therefore, K_u_ = 1 and ${\Delta {G}^{0}}_{u}=0$, thus indicating that the opposing tendencies balance each other, so ΔH_u_(T_m_) = T_m_ΔS_u_(T_m_). As a consequence, the temperature dependence of the ΔG_u_ may be written as follows:

7 $$\Delta {G}_{u}\left(T\right)=\Delta {H}_{u}\left({T}_{m}\right)\cdot \frac{{T}_{m}-T}{{T}_{m}}+\Delta {C}_{p,u}\cdot \left\{T-{T}_{m}-T\cdot \text{ln}\left(\frac{T}{{T}_{m}}\right)\right\}$$

Interestingly, due to “entropy-enthalpy compensation,” Gibbs free energy of unfolding is much less affected by temperature change than enthalpic and entropic components (Liu et al. 2000).

In the simplest case in which a ligand (L) binds specifically to a single site of the folded protein (F), a process can be described with the dissociation constant (K_d_) and the unfolding of such a complex depends on the ligand concentration as follows:

8 $${K}_{d}=\frac{\left[P\right]\cdot [L]}{[PL]};\quad {K}_{u,0}=\frac{\left[U\right]}{[F]};\quad {K}_{u}(L)=\frac{[U]}{(\left[P\right]+\left[PL\right])}=\frac{{K}_{u,0}}{1+\left[L\right]/{K}_{d}}$$

where [P], [L], and [PL] indicate the population of free folded protein, free ligand and their complex, respectively. In the presented case, the total population of folded protein and ligand equals [F] = [P] + [PL] and [L]_0_ = [L] + [PL], respectively.

So, all ligands that bind preferentially to the folded form of the protein will stabilize this form, and the unfolding of the protein becomes less favorable when the ligand concentration increases. Furthermore, conversely, if the ligand binds preferentially to the unfolded protein form, it will shift the equilibrium towards an unfolded state. Such process can also be expressed in Gibbs free energy terms:

9 $${\Delta G}_{u}=-RT\cdot \text{ln}\left({K}_{u}\right)=\Delta {G}_{u,0}+RT\cdot \text{ln}(1+\frac{\left[L\right]}{{K}_{d}})$$

It should be highlighted that K_d_ and K_u_ are likewise highly dependent on temperature, just as K_u_,_0_ is. Combining Eqs. 9 with 7 leads to the interpretation of a concentration-dependence of the so-called thermal shift, ΔT_m_ = T_m_—T_m,0_, as a rough estimate of the binding affinity (i.e., dissociation constant), where, however, temperature-dependences of the thermodynamic parameters (ΔH_u,0_ and K_d_) are commonly neglected:

10 $$\frac{\Delta {T}_{m}}{{T}_{m,0}}=\pm \frac{R{T}_{m,0}}{\Delta {H}_{u,0}({T}_{m})}\cdot \text{ln}(1+\frac{\left[L\right]}{{K}_{d}({T}_{m})})$$

The sign “ + ” in the latter equation is valid when a ligand binds to the folded target, while the “- “ one reflects events when a ligand preferably binds to an unfolded protein.

Some traps in applying DSF for ligand screening arise from the abovementioned fundamental thermodynamic relations. Firstly, several assumptions that are not necessarily valid for all tested systems must be made. Among them is the appropriate folding/unfolding model (e.g., reversibility, two-state/three-state model), but also ΔC_p_ negligibly varying with temperature. Moreover, ligands can simultaneously bind to the folded and unfolded states of the target, each of which interactions will have an opposite effect on the T_m_ shift. Physico-chemical properties of tested compounds also affect the stabilization effect due to the entropy of mixing. Moreover, compounds that bind at multiple sites must be appropriately analyzed. Finally, the temperature variation of the ligand’s binding enthalpy and entropy may differ. So, enthalpy-driven ligands commonly display lower ΔT_m_ than those whose binding is entropy-driven, even though they have the same binding affinity at the reference temperature (Deleeuw et al. 2021). In extreme cases, a flexible hydrophobic ligand, characterized by a large unfavourable entropy change upon binding, may apparently destabilize the target protein (decrease of T_m_) since, at higher temperatures, it binds stronger to an unfolded protein despite the reasonable binding affinity to the folded protein at physiological temperature. (Winiewska-Szajewska et al. 2019).

**Fig. 4:**
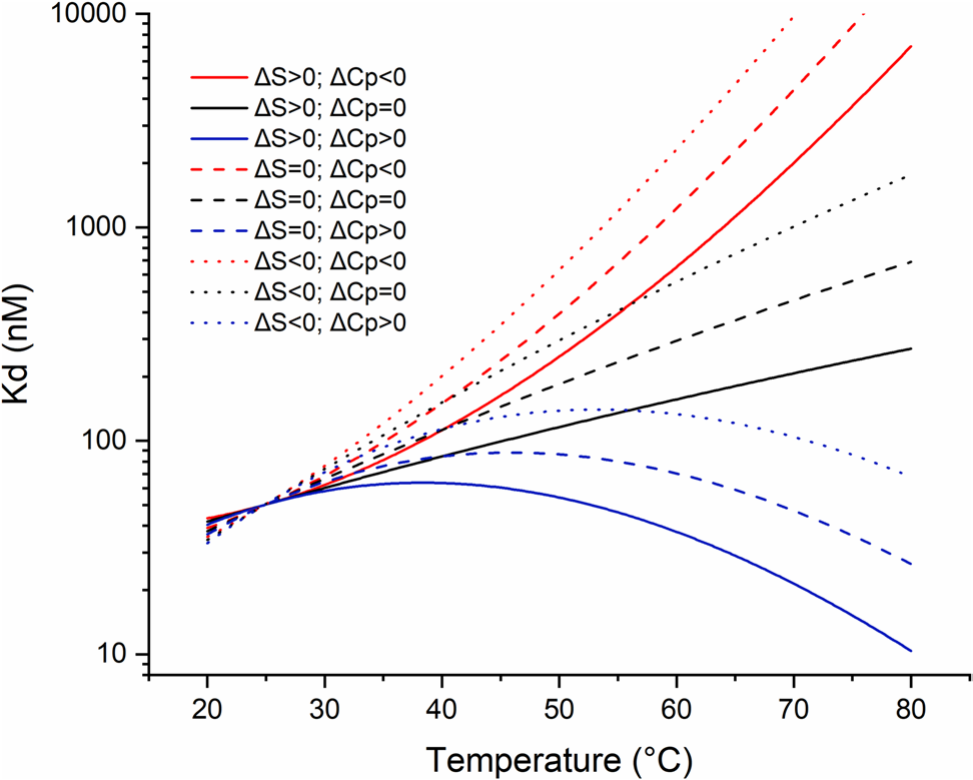
Simulation of binding affinity variation with temperature. Graph presents how Kd(25 °C) = 50 nM change with temperature depending on heat capacity change (assuming that ΔC_p_ does not vary with temperature) and entropy of binding. Blue lines show events with positive ΔC_p_ = 2 kJ/mol_,_ while black lines are for ΔC_p_ = 0 and red for negative ΔC_p_ = −2 kJ/mol. Solid lines are for positive entropy change during the binding ΔS(25 °C) = 50 J/mol; dashed lines are for ΔS(25 °C) = 0, and dotted lines are for negative entropy ΔS(25 °C) = −50 J/mol

The temperature dependence of the binding affinity reflects the thermal evolution of thermodynamic parameters characterizing protein–ligand interaction. In the simplest model, the evolution of ΔS_d_ and ΔH_d_ is described by Eqs. 4 and 5, respectively, while ΔC_p_ was assumed temperature-independent. In such a case, the thermal evolution of binding affinity substantially depends on the entropy-enthalpy balance, even neglecting heat-capacity change upon ligand binding (ΔC_p_ = 0, black lines in Fig. 4). Interestingly, ΔC_p_ contribution qualitatively affects the shape of the K_d_(T) relationship. Thus, the relation, being a flat line when ΔC_p_ = 0, becomes convex when ΔC_p_ < 0 and concave when ΔC_p_ > 0). The latter indicates that binding affinity may increase with temperature in particular conditions (see blue lines in Fig. 4). In general, the sign of ΔC_p_ reflects the balance of apolar (negative contribution) and polar (positive contribution) surfaces buried upon binding (Perozzo et al. 2004). Thus, the value is generally negative for moderately hydrophobic ligands or when the binding is coupled with conformational changes (Vega et al. 2016). However, for highly polar (or charged) ones, the positive ΔC_p_ could be observed, as was reported for metal binding by myoglobin (Kaur et al. 2017), DNA (Wu et al. 2005) or cap binding by eIF4E (Niedzwiecka et al. 2002). Interestingly, positive changes were also reported when peptides bind to the unfolded form of protein, enhancing such binding mode relative to the natural folded target (Varadarajan et al. 1992). The latter explains mentioned at the beginning phenomenon for ligands with close affinity to the same target at T_ref_ causing different ΔT_m_ and conversely, ligands with the same thermal stabilization differing in their binding affinity at T_ref_. Analogously, the same ligand may differently stabilize closely related proteins (e.g., carrying single-point mutations) differing in T_m_, despite the binding affinities at T_ref_ being identical. Occasionally, ligand binding may decrease the melting temperature, which effect may be observed when the ligand also binds to the unfolded form of the target. Due to different binding manner to folded and unfolded form, coupled with opposite heat capacity changes, one can imagine the possibility that at higher temperatures, binding to the unfolded form of the protein may dominate even if the binding is non-specific and without physiological significance at lower temperatures.

Another aspect is the difference in T_m_ monitored by intrinsic and extrinsic fluorescence. By design, fluorescent dye binds to the unfolded form of protein, shifting equilibrium in that direction and decreasing apparent melting temperature. Another reason for such a difference is simply monitoring slightly different events—in the case of natural fluorescence—changes in Trp and Tyr sidechains neighborhood, while in the second case, a partial exposure of the protein’s hydrophobic core to the solvent. Using other methods to measure thermal shift, like CD, which usually monitors the ellipticity change at 222 nm correlated with the α-helical content, may also result in slightly different T_m_ for the same reason. Thus, CD-based techniques monitor the conformation of the protein backbone, while Trp/Tyr fluorescence is generally affected by local changes in sidechain packing. However, differences in T_m_ measured using various methods may also indicate that the two-state mechanism of unfolding is not applicable (Luo et al. 1997; Casares-Atienza et al. 2011). Summarizing, in some particular cases, the discrepancies between thermal shifts determined with different approaches may be substantial; however, they usually differ only slightly (Chrabąszczewska et al. 2021).

It is crucial to consider technical limitations. Despite the efforts of many research groups to apply this method for direct binding affinity estimation (Matulis et al. 2005; Cimmperman et al. 2008; Bai et al. 2019; Hall 2019), it is essential to exercise caution. It is safer to consider this method just for high-throughput screening when some false positives and negatives are acceptable or for preliminary tests to be further confirmed by a quantitative method.

## Analytical methods to determine the binding affinity

### Microscale thermophoresis

**Fig. 5:**
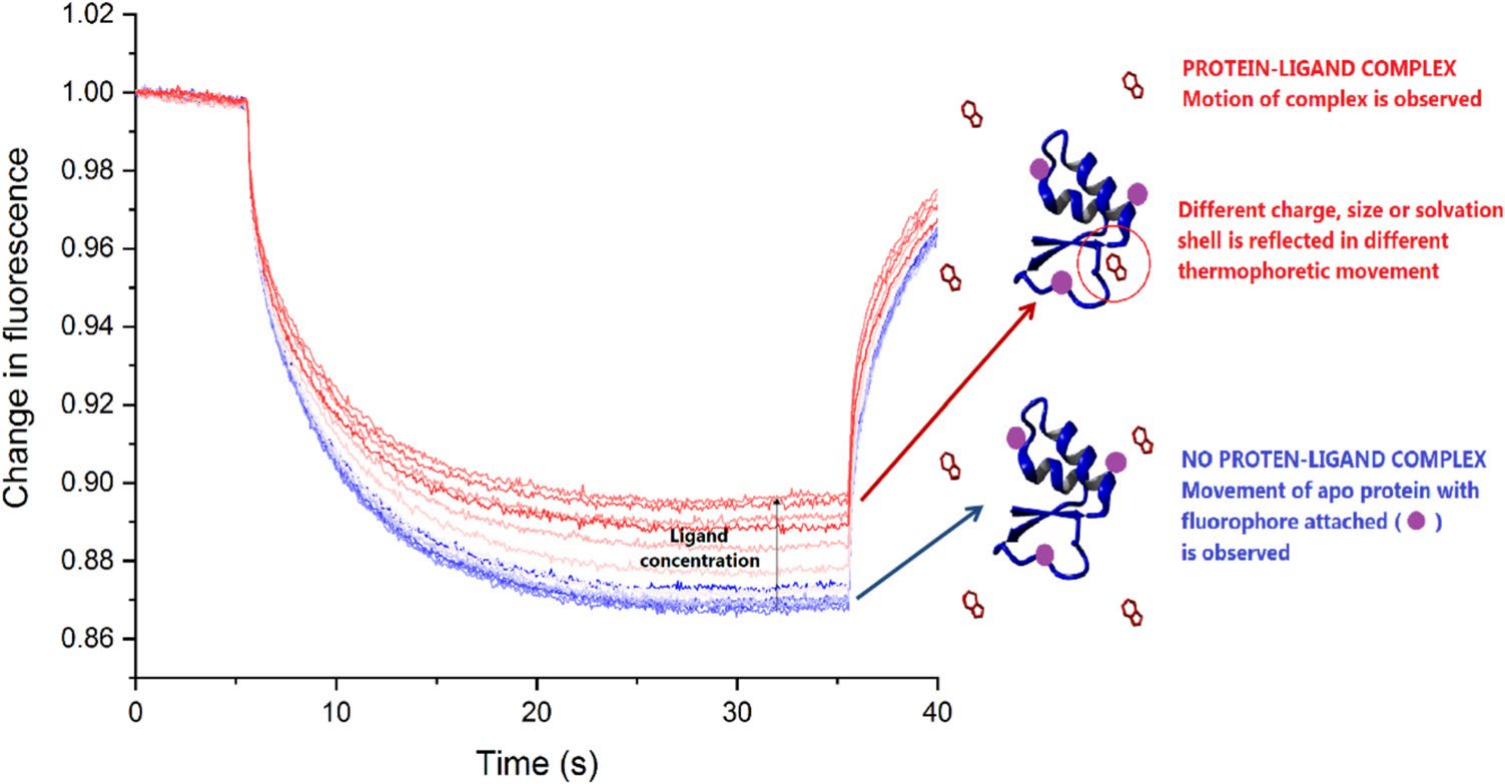
Microscale thermophoresis. A standard binding experiment with the cartoon representing the bound and unbound proteins. The thermophoretic movement of a fluorescent molecule (blue trace; unbound protein) changes upon binding to a non-fluorescent ligand (red trace; protein–ligand complex), resulting in different traces because the complex has different charge, size or solvation

MST is a technique based on thermophoresis, the directed movement of molecules in a temperature gradient, the phenomenon discovered in 1856 by Carl Ludwig (Ludwig 1856) and further described by Charles Soret in 1879 (Soret 1879, 1880; Platten and CostesèQue 2004). Despite being a research subject for a long time, microscale thermophoresis, a tool for studying biomolecular interactions, was introduced and further commercialized in 2010 by Stefan Duhr and Philipp Baaske (Wienken et al. 2010). The general concept of this technique is that thermophoresis highly depends on different properties of molecules like size, charge, conformation, and solvation. In most cases, binding events change at least one of these properties, which should be reflected in molecules’ movement (Fig. 5). The motion is monitored with fluorescence when, at the same time, an infrared laser induces the temperature gradient. Unlike other methods, only one company supplies dedicated devices for thermophoresis measurements (i.e., NanoTemper Technologies GmbH).

#### Analyzing thermophoretic data

Like other methods, MST monitors some molecule features that change with the ligand binding. Therefore, the dissociation constant can be estimated by analyzing the response of the observed protein signal to ligand concentration. One can choose different analyzed signals depending on rational inspection of obtained results. It can be either the initial fluorescence (if ligand binding specifically affects this parameter) or relative fluorescence, the ratio of the fluorescence at a selected time after the IR laser has been turned on to the initial one. Analyzing the response ~ 1 s after turning on the IR laser can be interpreted as a case where observed changes are caused by the temperature dependence of fluorescence itself (referred to as a T-jump or temperature-jump) and not a thermophoretic event. In a standard MST experiment for protein–ligand binding, 16 serial dilutions of the non-fluorescent ligand to the solution of fluorescent protein at a fixed concentration should be prepared. MST instruments are always supplemented with dedicated software, usually sufficient to analyze the obtained data. Other independent software is already available (Scheuermann et al. 2016), but homemade procedures for analysis may also be applied (Winiewska et al. 2017). A few reviews already describe how the instrument works, demonstrate how to analyze data (Jerabek-Willemsen et al. 2011, 2014; Tso et al. 2018) and list the limitations and possible artefacts that can occur during the experiments (Scheuermann et al. 2016; LóPez-MéNdez et al. 2021). The great advantage of MST is that it is a very user-friendly software that guides step by step and allows to set up an experiment even for a completely inexperienced user. Additionally, improved extensive quality control allows the identification of the factors causing the most common problems and misinterpretations.

### Isothermal titration calorimetry

ITC is a technique that directly measures the heat released or absorbed upon mixing reagents. The first isothermal calorimetric device was built as early as the eighteenth century (Lavoisier and De 1921), while the titration device enabling quantitative determining of the heat of reactions was described in 1959 (Schlyter and Sillen 1959). Since then, this method has been extensively developed (Izatt et al. 1974; Christensen et al. 2004; Lubbers and Baudenbacher 2011), leading to high precision and sensitive commercial calorimeters. The devices enabling high-throughput screening are also available (e.g., Automated MicroCal PEAQ-ITC from Malvern Panalytical). ITC has been extensively used to study binding interactions for over 30 years, and the basis of the method, analytical procedures, and the strengths and weaknesses of this approach have been broadly revised since then (Wiseman et al. 1989; Freire et al. 1990; Doyle 1997; VeláZquez-Campoy et al. 2004; Freyer and Lewis; 2008; Tellinghuisen 2008; Abian et al. 2023; Bastos et al. 2023).

#### Analyzing calorimetric data

As should be expected for all commercial instrumentations, dedicated software allows the principal ITC data analysis, and software packages are broadly used (Keller et al. 2012; Zhao et al. 2015).

**Fig. 6:**
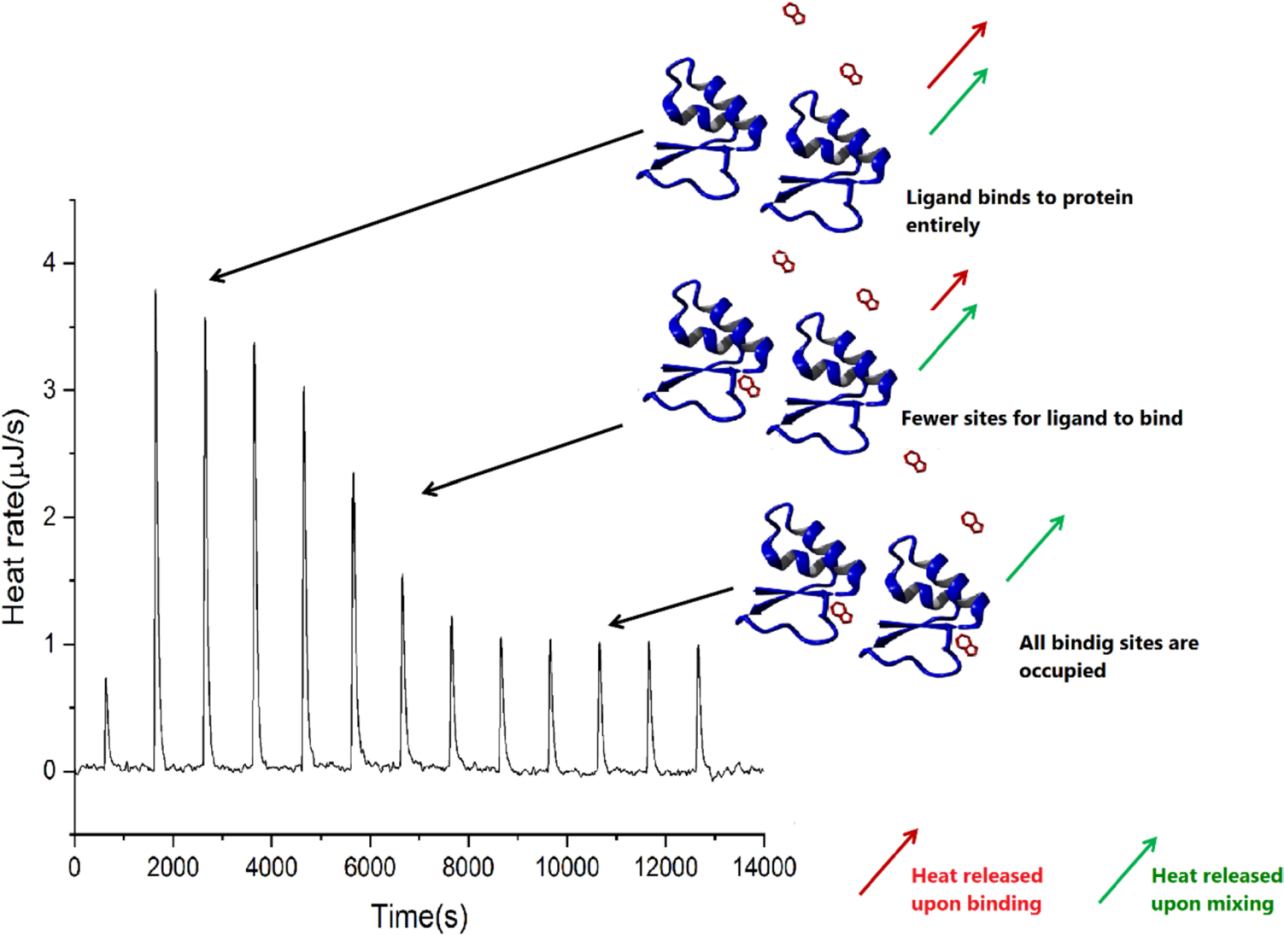
Isothermal titration calorimetry. Standard binding experiment with cartoon representing the bound and unbound protein and two dominating heat effects caused by ligand binding and mixing of components. The heat of each injection gradually reduces as free protein decreases upon ligand binding

Considering the single-site binding reaction, the thermodynamic interpretation is relatively straightforward when no additional effects occur. For each injection, the heat released (absorbed) is given by the following equation:

11 $${q}_{i}=V\cdot \Delta H\cdot \Delta {[PL]}_{i}$$

where q_i_ is the heat released or absorbed in each injection, V is the reaction volume, Δ[PL]_i_ is the change in bound ligand concentration between the (i)th and (i-1)th injections, and ΔH is the enthalpy of binding (Freire et al. 1990). Heat associated with each injection (q_i_) is determined from the area under each peak of the thermogram (Fig. 6). With the most commonly used experimental setup with constant aliquot titrations, the heat effect should be stepwise reduced, following trends in successive variations in binding equilibrium. In such a case, injection-induced changes in the population of the bound form progressively decrease after each injection. Finally, when saturation is reached, solely the consecutive effect corresponding to the heat of dilution, both protein and ligand, remains. However, the protein and ligand solutions should be prepared in identical buffers. Otherwise, the heat effects coupled directly with buffer mixing or secondary effects (e.g., pH or ionic strength variation) may contribute to the observed injection heat.

### Analyzing data—different binding models

Again, in the most straightforward single-site binding process, the dissociation constant is described by the relation of the concentration of free protein and ligand ([P] and [L]) to their complex ([PL]):

12 $${\text{K}}_{\text{d}}=\frac{\left[\text{P}\right]\cdot [\text{L}]}{[\text{PL}]}$$

For MST, as well as other methods where the observed signal changes upon the binding event, for each concentration ratio, we measure the contribution of signal from bound and unbound forms of protein according to Eq. 13:

13 $${F}_{obs}={f}_{a}\cdot {F}_{a}+{f}_{b}\cdot {F}_{b}$$

In such a case, the sum of fractions f_a_ and f_b_ equals 1, and both unbound and bound fractions can be described as follows:

14 $${f}_{a}=\frac{\left[P\right]}{\left[PL\right]+[P]};\quad{f}_{b}=\frac{[PL]}{\left[PL\right]+[P]}$$

Where the concentrations of free protein (P) and free ligand (L) can be estimated as the difference between the total concentration of protein and ligand ([P]_0_ and [L]_0_, respectively) and the actual concentration of formed complex (PL):

15 $$\begin{array}{cc}\left[P\right]={[P]}_{0}-[PL];& \left[L\right]={[L]}_{0}-[PL]\end{array}$$

The system of Eqs. (12)–(15) leads to the final equation applied in the NanoTemper software, in which the three unknown parameters (F_a_, F_b_, K_d_) are to be fitted.

16 $$F\left({L}_{0}\right)={F}_{a}+\frac{({F}_{b}-{F}_{a})}{2\cdot ({\left[P\right]}_{o}+{\left[L\right]}_{o}+{K}_{d}-\sqrt{{\left({\left[P\right]}_{o}+{\left[L\right]}_{o}+{K}_{d}\right)}^{2}-4\cdot {\left[P\right]}_{o} \cdot {\left[L\right]}_{o}}}$$

The above equations are also valid for sets of identical sites. A different approach is required for more complicated models. The supplier software additionally enables analysis of the cooperative binding with the adapted Hill model (Hill 1913) presented in the form:

17 $$F\left({L}_{0}\right)={F}_{a}+\frac{({F}_{b}-{F}_{a})}{1+{(\frac{EC50}{{[L]}_{0}})}^{n}}$$

However, such a model is only applicable for data with a single inflexion point. Moreover, instead of K_d_, the so-called Hill model allows only the determination of EC_50_. This value may be treated as a measure of apparent affinity in a particular experiment but may critically depend on the protein concentration used in the assay.

In ITC, the described above Eq. (11) also can be combined with Eqs. (14) and (15) leading to:

18 $${q}_{i}=V\cdot \Delta H\cdot ({f}_{b(i)}\cdot [{P]}_{0}-{f}_{b(i-1)}\cdot [{P]}_{0})$$

which should be further combined with (12) and (13) to give the final equation with two unknown parameters $\Delta H$ and K_d_ to be fitted (three if we analyze the number of sites, n):

19 $${q}_{i}=V\cdot \Delta H\cdot [{P]}_{0}\cdot \left(\frac{{\left[L\right]}_{i}}{{K}_{d}+{\left[L\right]}_{i}}-\frac{{\left[L\right]}_{i-1}}{{K}_{d}+{\left[L\right]}_{i-1}}\right)$$

This straightforward flow illustrates the basic concept of thermogram analysis. However, the final Eq. (19) should additionally be extended to account for two critical hardware-dependent changes that occur during repeated injections: protein concentration and volume changes. As a result, a correction should be performed to account for the fact that the protein concentration in the cell decreases with each injection, and some of the liquid will no longer be in the working volume. Of course, this is always incorporated in commercial software (ITC Data Analysis in Origin 2004; TA Instruments NanoAnalyze Software Getting Started Guide 2019) and should be in homemade analysis procedures (SokołOwska et al. 2009).

In the commercial software for ITC data analysis, other models considering different sets of binding sites are also implemented to study more complex interactions with even three distinct binding sites (Houtman et al. 2007; Gustchina et al. 2013). It is, however, crucial to understand what fitted parameters stand for in such models. While two sets of independent sites model allow fitting $\Delta \text{H}$ and K_d_ for both binding sites (analogously like for single site model/ single set of identical sites model described above), a different situation is in the case of models sometimes described in various software as “Sequential” or “Cooperative”. While some of them allow fitting microscopic parameters of binding (e.g., “Sequential Two Site” for NanoAnalyze (TA Instruments NanoAnalyze Software Getting Started Guide 2019)), for others, the fitted binding constants may not describe particular microscopic binding events hierarchy but are defined relative to the progress of saturation with no distinction to which sites are saturated (e.g. model “Sequential binding sites” for MicroCal software (ITC Data Analysis in Origin 2004) or “Cooperative” for NanoAnalyze (TA Instruments NanoAnalyze Software Getting Started Guide 2019)).

For MST, if more than one inflection occurred, more advanced models, for example, two or three sets of binding sites (e.g., independent or cooperative/sequential) or fraction sites, require dedicated analysis procedures (Winiewska et al. 2017; Tso et al. 2018) or unique approaches in planning experiments (Seidel et al. 2013).

As described above, the simplest model of ligand binding $P+L\leftrightarrows PL$ leads to the quadratic equation (Eq. 15) that can be formally resolved by radicals. The below-listed microscopic models of two independent (2i) or two sequential (2s) binding events can be applied to various protein–ligand systems, leading to the cubic equation, resolvable by radicals (method developed in XVI century by Scipione del Ferro, announced by Niccolò Fontana Tartaglia, published by Girolamo Cardano—Ars Magna, 1545 (Cardano and Spon 1968)):

- Two separated binding sites on a single protein molecule—2i (Wang and Jiang 1996; Tso et al. 2018).
- Two close/interacting binding sites on a single target molecule, either sequential—2s (Hyde et al. 2003) or independent—2i (Il'ichev et al. 2002).
- Two identical but interacting binding sites on a protein symmetrical dimer; a negligible fraction of protein monomer—2s (Bajor et al. 2016).
- Ligand binding at a dimer interface – 2s—applied formally in the reversed mode – 1 ligand (protein) + 2 target molecules (metal ions) (Bajor et al. 2016).

More complex systems that lead to the quartic equation can also be resolved by radicals (solved by Lodovico de Ferrari; published by Girolamo Cardano (Ars Magna, 1545 (Cardano and Spon 1968)).

- Three binding sites on a single target—all independent −3i (SokołOwska et al. 2009), two coupled combined with the third independent site—2s + i (Bajor et al. 2016), or three in sequence—3s (Yang et al. 2019).
- Protein dimerization coupled with ligand binding; both target forms bind a ligand (Levitzki and Schlessinger 1974).

Other systems can be occasionally analyzed (Fasano et al. 2005; Ascenzi and Fasano 2010), but they usually lead to higher-order polynomial equations (n > 4), which, according to the Abel-Ruffini theorem (Abel 1826), cannot generally be resolved in radicals. High-order binding polynomials could also be used in the data analysis; however, in such an approach, at each iteration of the main procedure of fitting the binding polynomial to the experimental data, the binding equilibria are to be resolved via an additional iterative numeric procedure (e.g., the Newton–Raphson method). However, a two-level iterative non-linear optimization requires special attention due to possible problems with the stability and convergence of the algorithms used.

The general approaches, such as numerous binding sites with unconstrained interactions between them (Herrera and Winnik 2013) or mentioned above general binding polynomials (Freire et al. 2009) have the primary advantage that data may be evaluated to obtain general information about system behaviour without making assumptions about the binding mechanism, which can sometimes be difficult to discern.

**Fig. 7:**
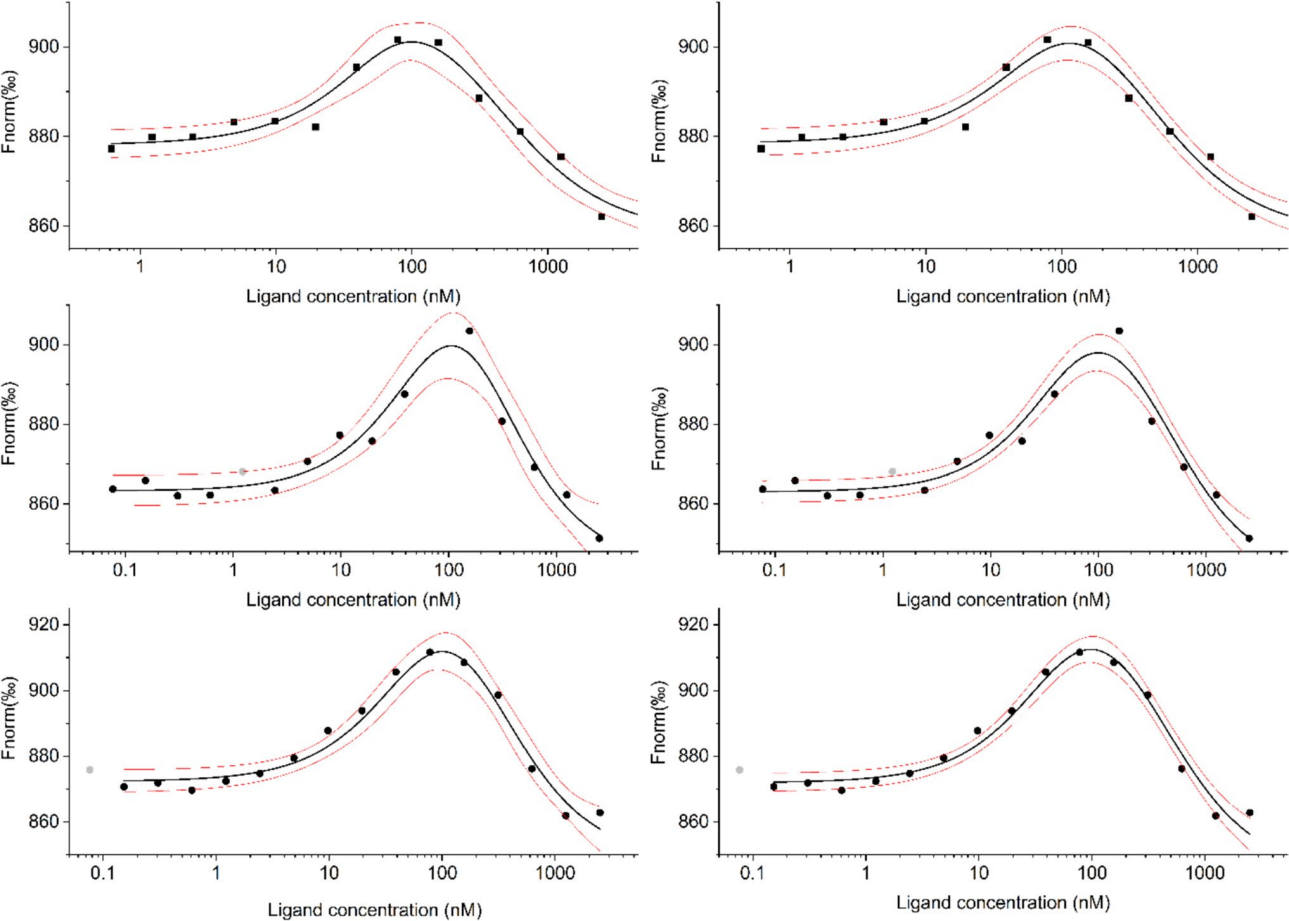
Comparison of MST data fitted separately (left panel) and globally (right panel). The example shown is the binding of 5,6-dibromo-1H-benzotriazole to hCK2α, results adapted from (Paprocki et al. 2021). Black circles show experimental data, grey ones indicate data removed from the analysis, thick black lines represent the fitted model of two sequential biding sites, and red ones border the 95% confidence limits for a particular model

It is crucial to recognize the need to analyze data globally for all these more complicated systems, which is generally a good practice even for simple single-binding-site experiments. Figure 7 compares the same MST data with two sequential binding sites modelled independently (left panel) and globally (right panel). In the presented example, the uncertainty of parameters fitted independently is up to two orders of magnitude higher than that of fitted using the global approach.

**Fig. 8:**
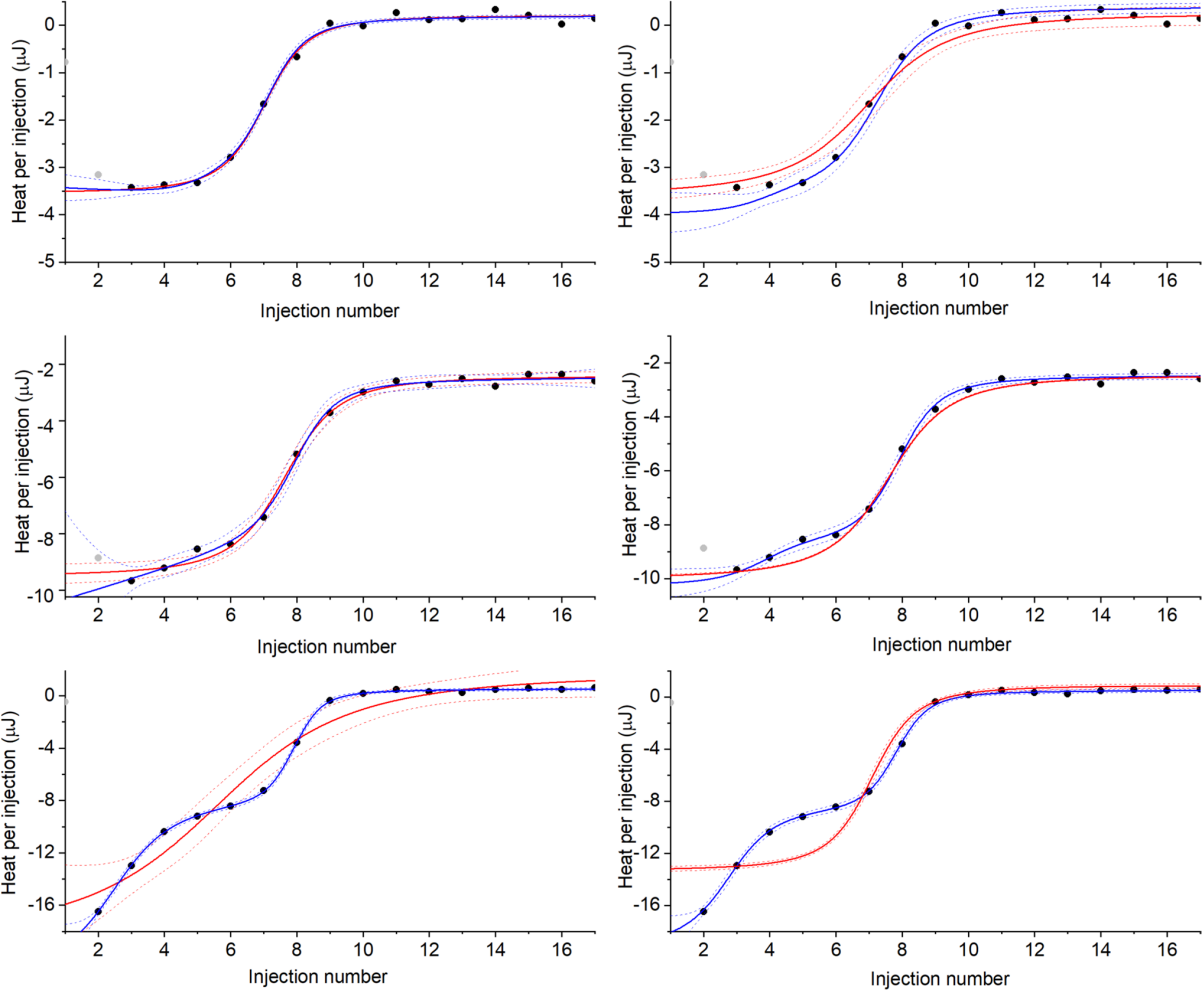
Comparison of ITC data fitted separately (left panel) and globally (right panel). The example shown is the binding of 4,7-dibromo-5,6-diiodo-1H-benzotriazole to hCK2α, results adapted from (Paprocki et al. 2021). Black circles show experimental data, grey ones indicate data removed from the analysis, thick blue lines represent the fitted model of two sequential binding sites, and red ones represent the fitted model of a single binding site. The dotted lines boarder the 95% confidence limits for a particular model

Especially for ITC, combining several experiments with slightly different concentrations is highly desirable to confirm if the selected model is correct (Freiburger et al. 2009; SchöNbeck et al. 2012). An example is in Fig. 8, where the left panel presents the ITC experiments with different concentrations analyzed individually, and the right one displays the same data analyzed globally. Two identical models were fitted in both cases—a single binding site (red) and two sequential binding sites (blue). In the presented scenario, a single binding site model may seem valid for lower concentrations; however, only a model of two sequential binding sites fits the data globally.

In the ITC, a combination of “normal” and “reverse” (where the protein states the titrant) experiments are used to obtain more accurate information about the obtained results and stoichiometry of a binding system (Dam et al. 2002; Winiewska et al. 2017). When possible, performing a “reverse” experiment in MST that analyzes the solution of fluorescent ligand at a fixed concentration, with serial dilutions of the protein, may as well be used to confirm the obtained results but also may give a better signal-to-noise ratio.

Sometimes, global analyses combine results from different methods, which can be done with homemade procedures (Xue et al. 2004; Herman and Lee 2009) or dedicated software (Zhao et al. 2015). Such an approach, however, presents some risks of favoring one type of experiment, so the selection of the appropriate method of weighting is crucial.

### ITC vs. MST

The differences in these methods occur already when planning the experiment, starting with the concentration range chosen for the macromolecule and the ligand. For MST experiments, the choice of concentrations is more manageable even if we roughly know the studied system. Generally, the fixed macromolecule concentration (the fluorescence of which is monitored) should be lower than an expected K_d_. However, choosing a concentration minutely higher or of the same order as the dissociation constant will not foreclose obtaining reliable results. However, the observed signal variation will also reflect the stoichiometry of the binding process. The maximal ligand concentration should exceed K_d_ by at least one order, sufficient for the saturation of the protein–ligand complex. However, when binding affinity is unknown, a high excess of the ligand is recommended to extend the tested concentration ranges. For ITC experiments, choosing reasonable concentrations for the macromolecule and the ligand is more complicated since there are far more factors for consideration. First of all, the described by Wiseman et al. so-called c value (Wiseman et al. 1989), which in the case of a 1:1 binding is defined as the ratio of total macromolecule concentration in the cell (named as [P_0_] here) to dissociation constant (K_d_), should be in the appropriate range to credible estimate all binding-related thermodynamic parameters (to obtain sigmoidal curve). Moreover, unbound and fully saturated states should be sampled to obtain reliable results, so the ligand concentration has to be preoptimized based on stoichiometry, either predicted or estimated. Otherwise, only the binding affinity could be reasonably estimated for hyperbolic-shaped titration isotherms, while uncertainties of strongly correlated binding enthalpy and stoichiometry values are highly biased.

Next, the planned concentrations should result in measurable enthalpy change, which may hardly be met, especially for entropy-driven binding events—the heat released/absorbed in each injection must be higher than the noise. The latter can be overcome by increasing the volume and reducing the number of injections in the titration experiment.

In both MST and ITC experiments, the aggregation at higher concentrations may be the limitation. In the calorimetric method, if either titrant or analyte is aggregating, the obtained results become unreliable. Contrarily, the pseudo-titration curve obtained in thermophoresis could be cut to omit “titrant” aggregates occurring at higher concentrations, and the only difficulty is that saturation may not be achieved. Another thing is that in the ITC experiment, protein and ligand concentrations must be accurately known, and any bias contributes to systematic errors in stoichiometry and enthalpy. In MST experiments, concentrations are also important but less crucial for a protein, especially when its concentration is lower than K_d_. For both methods, simulation algorithms can help choose suitable concentration ranges. However, MST is more robust in handling mistakes when planning this aspect.

Another aspect is the time of data acquisition. ITC calorimeter measures the heat change in real-time when titrant solution is injected into the calorimetric cell. In MST experiments, the ligand and protein solutions are mixed at least several minutes before the data acquisition. This difference implies some consequences. ITC is more convenient for measuring reaction kinetics (Egawa et al. 2007; Wang et al. 2020); however, the obtained dissociation constants may be biased for slow-binding ligands (or in extreme cases, even not measurable) or due to additional time-dependent events occurring upon injection of the concentrated titrant into the solution, including induced conformational changes, oligomerization state, or ligand nano-aggregates dissolution (Winiewska et al. 2017). In the MST experiment, the equilibrium state is commonly achieved. Moreover, buffer mismatch heat effects may affect calorimetric results more significantly than in the case of thermophoresis.

ITC is a label-free method, while MST has two variants: one where intrinsic fluorescence is monitored and the second with several possible labelling approaches. The variant without labelling is limited to systems where only one binding partner exhibits natural fluorescence. Therefore, MST with labelling is more common; however, like for every method that requires labelling, there is a risk that fluorescent tags could interfere with ligand binding. Fortunately, it is possible to catch such situations by repeating experiments using different labelling strategies.

If the experiments were performed correctly, both methods would not yield false positive results. However, some artefacts may occur in both techniques, and there is still a risk of misinterpretation. ITC, while more informative, is also more prone to that type of occurrence as many other effects are reflected in heat change. Moreover, integrated quality control in MST, including capillary scans or time traces, allows the detection of sticking and aggregation/precipitation effects.

Even using the latest highly sensitive, low-volume calorimeters, the time and sample consumption are still significantly higher than for MST. The latter method is also more robust to suboptimal experimental setups and much easier to optimize for poorly defined systems. ITC, on the other hand, is very sensitive and provides essential information about the nature of the interaction: not only the binding stoichiometry and the affinity but also the formal thermodynamic parameters associated with the binding (enthalpy, entropy, and Gibbs free energy) that are very useful for further optimization of compound structure for the drug candidate.

After analyzing the above differences, combining both methods (starting with MST) is a great way to facilitate optimization, provide better insight into the binding, and catch some discrepancies and artefacts.

## The necessity of confirmation by an independent method

We commonly find in the literature that two methods are used when results are ambiguous or there is a need to supplement one method because of its limitations. Such an approach should be a standard procedure whenever it is applicable.

We previously showed how results from ITC and MST for benzotriazole derivatives binding to CK2 differ by an order of magnitude, and the detailed analysis of this difference allowed us to find an unbiased result (Winiewska et al. 2017). These two methods were also used in screening Myricetin derivatives for inhibitors of Cucumber Mosaic Virus 2b (Wang et al. 2022). While the authors were focused on the in vivo results, they did not comment on the discrepancies between ITC and MST data, possibly resulting from limitations in experimental setup and analysis of calorimetric experiments. Another example of using ITC and MST methods is the interaction of the androgen receptor-DNA binding domain and antiandrogen SBF-1 (Elgehama et al. 2021). Interestingly, the authors decided not to compare the results obtained by these two methods, while they differ by three orders of magnitude. However, the dissociation constants calculated from MST data seem contradictory with the presented MST curves, which is putatively a reason for discrepancies. On the other hand, in studies concerning molecule MYCMI-6 as an inhibitor of MYC: MAX interaction, two methods, SPR and MST, were used. While both methods yield similar results, the authors estimated the dissociation constant solely based on SPR data (Castell et al. 2018). As another example, authors that presented novel glucopyranoside derivatives as potential antiviral agents against tobacco mosaic virus using three different approaches, i.e., fluorescence spectroscopy, ITC and MST, obtained similar estimates of binding affinities, so their results can be considered consistent (Chen et al. 2015a). In studies of Kanzaki et al. on binding CRL1101 to RelA protein, immobilization of RelA, necessary for SPR, probably was interfering with ligand binding; therefore, MST and ITC techniques were applied and also consistent results were obtained (Kanzaki et al. 2021). As a somewhat different example, in studies concerning the connection between binding metal ions and ligands to integrin I domains, two methods, SPR and ITC, were used, although slightly different objects were investigated using each method. Nevertheless, differences in obtained results were analyzed, leading to valuable conclusions (Vorup-Jensen et al. 2007). Sometimes, even more complementary methods are applied. In the paper of Narczyk et al. (Narczyk et al. 2021), due to some limitations, different binding events were followed with the aid of different methods, i.e., phosphate binding to PNP was measured with the aid of fluorescence, CD and MST titrations and nucleoside analogue binding was measured with the aid of MST and ITC. While some differences occurred in results obtained using a broad pool of methods, the authors succeeded in interpreting this discrepancy. An excellent example of the importance of the validation of the binding with the aid of other methods is the studies on the interaction of Mycobacterium tuberculosis ribosomal protein S1 (RpsA) with pyrazinoic acid (Vallejos-SáNchez et al. 2020). The authors used NMR, ITC, and EMSA assay to prove that RpsA does not interact with pyrazinoic acid by thus overturning previously published results suggesting strong interaction (Shi et al. 2011; Yang et al. 2015). Meanwhile, Jecklin et al. compared the nESI-MS, SPR and ITC methods for the assessment of sulfonamide inhibitors binding affinity to the human carbonic anhydrase I, showing that while for some compounds, the agreement between the three methods is almost perfect, it is poor for the others. The possible reasons for such a disagreement are thoroughly discussed (Jecklin et al. 2009).

These are just a few instances demonstrating that, according to best practices, obtained results characterizing binding affinities should be validated by another biophysical method whenever possible, even if the single-method results appear reliable.

## Recent applications of DSF combined with other biophysical methods in ligand screening

The idea of combining different biophysical methods to screen a library of compounds has already been suggested several times. Aside from crystallography, it typically starts with DSF, and the found hits are then validated using other methods.

The work of Guo et al. is an excellent example of applying DSF combined with another biophysical method for ligand screening (Guo et al. 2021). They performed a screening against about 5000 compounds synthesized by their group to find compounds interacting with SET domain bifurcated histone lysine methyltransferase 1. Only one screened compound caused a thermal shift exceeding 1.5℃, and its binding was further confirmed with the aid of ITC. In the next step, the authors solved the crystal structure of the complex with this ligand and optimized compounds based on the obtained structure. Almost all newly synthesized compounds exhibited stronger binding affinity, as was confirmed by DSF and ITC and, for the most promising one, also with the SPR. All obtained results were consistent and further confirmed in vivo.

Researchers from Cambridge University proposed a slightly different approach for ligand screening with DSF (Mashalidis et al. 2013; Silvestre et al. 2013). They presented the procedure with preliminary DSF screening, further validated by ^1^H NMR spectroscopy followed by ITC and X-ray crystallography to characterize binding for the most promising compounds. Their strategy involved two screening methods rather than two ways for estimating binding affinity, which is very useful in the event of many positive results. Another approach to validate DSF results was presented by Gradl et al., where catalytic inhibition of hits from screening was tested. Next, the authors crystalized the most promising compound, optimized it to synthesize new ligands, and finally applied ITC and SPR to confirm affinity for the most promising one (Gradl et al. 2021).

Differential scanning fluorimetry is not only used as a first preliminary step but sometimes also as a complementary method. McCoy et al. employed DSF and ITC to demonstrate that molecules described previously as β-catenin's ligands do not bind to it in vitro, contrary to claims based on in vivo investigations (Mccoy et al. 2022). Another example of such an approach was studies of sulfonamides binding to human carbonic anhydrase XII (Jogaitė et al. 2013). Redhead and collaborators combined DSF with SPR to test ligands of the kinase p38α and several known pan-assay interference compounds. They estimated the dissociation constant based on DSF data, compared it to the one determined using SPR, and found good agreement between these two (Redhead et al. 2015). The interactions between newly designed compounds and carbonic anhydrase II were studied using DSF, ITC, and SPR, and the resulting affinities were compared (Rogez-Florent et al. 2014). The authors demonstrated that ITC and SPR results agreed almost perfectly, while dissociation constants estimated from DSF data slightly differed, which was unsurprising, given the assumptions and simplification made for latter calculations. Nonetheless, all three methods showed the same affinity scale and correctly ordered all compounds.

Because of low sample consumption, MST and DSF can often be found together as complementary methods besides ligand screening. As examples from our backyard, these two methods were used to compare the affinity of hNudt16 towards a set of substrates (Chrabąszczewska et al. 2021) or benzotriazole derivatives’ affinity towards hCK2α (Winiewska et al. 2015). In both these studies, the dissociation constants obtained from MST and DSF data agreed. The same approach was followed in studies on rigosertib, a styryl-benzyl sulfone, and its interaction with the RBDs domain of RAF kinases, which was confirmed by ΔTm and further assessed by MST-derived Kd (Athuluri-Divakar et al. 2016). In a slightly different example, MST was used to compare the binding affinity of various multi-granulin domain peptides and full-length progranulin to Pro-cathepsin D. DSF was used to analyze the destabilization effect of these peptides on Pro-cathepsin D (Butler et al. 2019). As described above, one should be, however, careful with the interpretation of protein destabilization in case of binding associated with a large entropic contribution, like for at least partly hydrophilic peptides (reduction of degrees of freedom for peptide in solution versus in complex), because such effect may have strong anomalous thermodynamic contribution due to positive heat capacity change. That is also why DSF is not a reliable method to directly compare the affinities of peptides with different lengths and structures. However, it can still be reasonable to use DSF in the case of similar peptides like it was done by Molledo et al. to compare tripeptides binding to a proton-dependent oligopeptide transporter (Martinez Molledo et al. 2018). Indeed, in this case, the measured thermal shifts agreed with the MST data. On the other hand, studies on inhibitors of the programmed cell death 1 (PD-1)/programmed cell death-ligand 1 (PD-L1) interaction (Ganesan et al. 2019) proved how different sizes and physicochemical properties of compounds lead to different thermal shifts. In that case, the authors’ awareness and supplementation of DSF results with SPR and MST averted misinterpretation.

Combining DSF with two (or more) quantitative methods for ligand screening might seem unnecessary, as further in vivo steps are always required to confirm the mode of action. However, when tested compounds differ in binding affinity very slightly and/or ligands will be further optimized, the most accurate binding affinities, probably with some additional thermodynamic information, are needed. That was the case for studying dihydropteridinone and pyrimidodiazepinone kinase inhibitors against eight different bromodomain and extraterminal (BET) proteins (Karim et al. 2021), where not only DSF, MST, and ITC were applied, but also additional (qPCR)-based assay to correctly analyze differences in binding affinities. In 2021, we compared four ligands with marginally different affinities, and only since all applied methods (i.e., DSF, MST, ITC and activity assay) gave similar results, we decided to analyze these differences (Paprocki et al. 2021).

Although each of the suggested methods is widely used, and there are plenty of instances of their application in the literature, very few studies employ all three approaches simultaneously. Table 2 presents such examples with the described purpose of using these three methods and other methods applied for this purpose.

**Table 2:** List of studies, in which all three methods: DSF, MST, and ITC were used

Studied target	Purpose	Other methods applied	Reference
Bromodomain-containing protein 4 (BRD4)	Characterize binding with a series of known and newly identified inhibitors	qPCR assay	(Karim et al. 2021)
The catalytic subunit of protein kinase CK2 (CK2α)	Screening for peptide inhibitors	Ligand-observed MS; NMR	(Winiewska-Szajewska et al. 2019)
Linker between the PYK2 kinase and FAT domains (KFL) in PYK2	Confirming and characterizing binding with calmodulin	SEC, AUC, and a number of additional biophysical approaches for different purposes	(Momin et al. 2022)
Bromodomains of EP300, CBP	Characterize binding with a series of intermediates and derivative of CCS1477		(Shendy et al. 2024)
FMDV capsids	Characterize binding with divalent transition metal ions	MALS	(Lin et al. 2021)
FAT domain from FAK and CH domain of α-parvin	Testing the affinity of the computationally predicted LD motifs		(Alam et al. 2020)
Intestinal mucin MUC2	Confirming copper binding	X-Ray, UV/Vis-competition titration,	(Reznik et al. 2022)
Bromodomain of BRD9	Screening for inhibitors	SPR, NMR, X-Ray	(Martin et al. 2016)
Elongation factor P (EF-P) from *Acinetobacter baumannii*	Confirming c-di-GMP binding		(Guo et al. 2022)
LMTK3 kinase domain	Screening for inhibitors	TR-FRET, kinase assay, CD	(Ditsiou et al. 2020)
Variants of the catalytic subunit of protein kinase CK2 (CK2α)	Characterize binding with the series of benzotriazoles		(Winiewska-Szajewska et al. 2024)
Aldehyde dehydrogenase 1A3 (ALDH1A3)	Confirming YD1701 binding	* CETSA instead of TSA	(Duan et al. 2022)

Of these publications, only two use all three methods for screening. As in the paper of Ditsiou et al., the 28,716 compound screening against the LMTK3 kinase domain was done mainly with the biochemical assay based on TR-FRET. A single hit – C28 compound was then confirmed with the aid of DSF and CD thermal shift, and additionally, ITC and MST were used to study the ternary system with this compound and HSP90α/CDC37 (Ditsiou et al. 2020). On the other hand, in Martin et al. work, the 1700 compounds were screened against the bromodomain of BRD9 using three parallel screening methods: DSF, SPR, and MST. It is worth mentioning that this is one of the first examples of using MST for screening. After the first screening, 77 hits were validated with the aid of NMR, and then 55 of these compounds were successfully soaked into the crystals. Next, detailed optimization of compound structure and additional rounds of studies were applied, and finally, the binding parameters were measured for two compounds with the aid of ITC (Martin et al. 2016). In the last example, 640 peptides were screened against hCK2α with affinity chromatography coupled with MS, and 9 hits were further studied simultaneously with NMR and DSF. Finally, one compound was validated and characterized with the aid of MST and ITC (Winiewska-Szajewska et al. 2019). Although one can argue that none of the presented examples accurately represent the proposed procedure, the fact that most of the publications presented in the table were written in the last 4 years allows us to assume that there is an increasing interest in combining these methods, most likely also in screening. DSF itself has been widely used for high-throughput screening of libraries ranging from several dozen (Chauhan et al. 2016, 2019; Winiewska-Szajewska et al. 2021) to several hundred (Fedorov et al. 2007) or even thousands (Gradl et al. 2021; Guo et al. 2021) of compounds. The other two methods are standardly used to study interactions with inhibitors. Therefore, combining these methods seems to be a natural and rational course of events.

## Conclusions

Differential scanning fluorimetry (DSF) is a powerful, high-throughput biophysical technique applicable in the early stages of drug discovery. The most significant advantages of DSF are accessibility, short measuring time, and relatively low cost. However, this method is susceptible to false positives and negatives, so another quantitative method should confirm at least positive screening hits.

Isothermal titration calorimetry (ITC) and microscale thermophoresis (MST) are widely used and may be treated as complementary. MST is rapid (15 min for the single experiment) and precise method with the dissociation affinity covering the pM to mM range. The great advantage of this method is the fast and flexible assay setup and optimization. ITC, on the other hand, is a powerful technique providing additional thermodynamic parameters concerning the binding that can be very useful in further optimizing effective ligands.

While we do not argue that different combinations may be similarly effective, our experiences let us propose screening with the aid of differential scanning fluorimetry followed by microscale thermophoresis, further confirmed/supplemented with the isothermal titration calorimetry as an easy, fast, robust and effective way to find promising compounds.

## Data Availability

No datasets were generated or analysed during the current study.
